# A Small Molecule SMAC Mimic LBW242 Potentiates TRAIL- and Anticancer Drug-Mediated Cell Death of Ovarian Cancer Cells

**DOI:** 10.1371/journal.pone.0035073

**Published:** 2012-04-25

**Authors:** Eleonora Petrucci, Luca Pasquini, Manuela Bernabei, Ernestina Saulle, Mauro Biffoni, Fabio Accarpio, Simone Sibio, Angelo Di Giorgio, Violante Di Donato, Assunta Casorelli, Pierluigi Benedetti-Panici, Ugo Testa

**Affiliations:** 1 Department of Hematology, Oncology and Molecular Medicine, Istituto Superiore di Sanità, Rome, Italy; 2 Department of Surgery Pietro Valdoni, Policlinico Umberto I, University of Rome “Sapienza”, Rome, Italy; 3 Department of Obstetrics and Gynaecology, University of Rome “Sapienza”, Rome, Italy; Enzo Life Sciences, Inc., United States of America

## Abstract

**Background:**

Ovarian cancer remains a leading cause of death in women and development of new therapies is essential. Second mitochondria derived activator of caspase (SMAC) has been described to sensitize for apoptosis. We have explored the pro-apoptotic activity of LBW242, a mimic of SMAC/DIABLO, on ovarian cancer cell lines (A2780 cells and its chemoresistant derivative A2780/ADR, SKOV3 and HEY cells) and in primary ovarian cancer cells. The effects of LBW242 on ovarian cancer cell lines and primary ovarian cancer cells was determined by cell proliferation, apoptosis and biochemical assays.

**Principal Findings:**

LBW242 added alone elicited only a moderate pro-apoptotic effect; however, it strongly synergizes with tumor necrosis factor-related apoptosis inducing ligand (TRAIL) or anticancer drugs in inducing apoptosis of both ovarian cancer cell lines and primary ovarian cancer cells. Mechanistic studies show that LBW242-induced apoptosis in ovarian cancer cells is associated with activation of caspase-8. In line with this mechanism, c-FLIP overexpression inhibits LBW242-mediated apoptosis.

**Conclusion:**

LBW242 sensitizes ovarian cancer cells to the antitumor effects of TRAIL and anticancer drugs commonly used in clinic. These observations suggest that the SMAC/DIABLO mimic LBW242 could be of value for the development of experimental strategies for treatment of ovarian cancer.

## Introduction

Cancer is very complex multistep disorder involving the progressive accumulation of genetic and epigenetic abnormalities, which ultimately lead to the transformation of normal cells into malignant cells displaying the essential properties of cancer: resistance to apoptotic mechanisms, independency from growth signals, insensitivity to negative growth signals, invasive and metastatic capacities, unlimited replicative potential and sustained angiogenesis [1]. Among these various properties of cancer cells, the resistance to apoptosis certainly plays a very relevant role in tumor development and progression. The ability of cancer cells to evade apoptosis is related to various biochemical properties of these cells, and particularly, to the up-regulation of antiapoptotic genes such as certain members of the Bcl-2 family of proteins and members of the Inhibitor of Apoptosis (IAP) family of proteins [2]. Particularly, three lines of evidence support a role for IAP proteins in cancer: (i) elevated expression levels of IAP proteins, particularly XIAP, c-IAP1 and c-IAP2, in a number of human cancer types correlate with tumor grade and prognosis [3]; (ii) a number of *in vitro* and *in vivo* studies have shown that downregulation of XIAP or c-IAP1 by various agents results in sensitization of cancer cells to chemotherapy- and gamma irradiation-induced apoptosis [4]; (iii) the chromosomal region 11q21-q23 containing c-IAP1 and c-IAP2 genes is subject to chromosomal amplification in various tumors [3], [4].

IAPs, and particularly c-IAP1, c-IAP2 and X-linked IAP (XIAP), function to inhibit apoptosis by preventing activation of caspases-8 or inhibiting the activity of caspases-9, -3 and -7, respectively [5],[6]. C-IAP1 and c-IAP2 possess an E3 ubiquitin ligase domain that promotes proteasome-dependent degradation of c-IAP1 and c-IAP2 [7]. The activity of IAPs is antagonized by SMAC/DIABLO (second-mitochondria-derived activator of caspases/direct inhibitor of apoptosis-binding protein with low pI) that, after release from mitochondria in response to apoptotic triggering, undergoes maturation and cleavage of its N-terminal region, with consequent exposure of the AVPI sequence [8]. This tetrapepetide binds XIAP and competes with the same binding sites that are involved in the interaction with caspases [9]. Through this mechanism, SMAC/DIABLO prevents the sequestration of caspases by IAPs, thus facilitating the apoptotic pathway. Since the AVPI sequence is able to promote apoptosis, compounds able to mimic this tetrapeptide, collectively known as SMAC-mimetics, have represented the objective of intensive research efforts and several of these agents have been developed during these last years [10]–[15].

It is important to note that a deregulation of IAPs may contribute to tumor development not only through caspases inactivation, but also through different mechanisms not dependent on caspases inactivation. Thus, a recent study clearly showed that: XIAP contributes to metastasis *in vivo* and cell invasion *in vitro*, independently of caspases binding and inhibition; XIAP in complex with survivin drives the activation of NF-κB to promote cell invasion and metastasis; c-IAP1 and c-IAP2 are also involved in cancer cell invasion [16].

Thus, inactivation of IAPs, particularly when combined with other treatments (such as chemotherapic drugs, death ligands including TNF-alpha and TRAIL), results in the death of most tumor cells, at least under tissue culture conditions [11], [13], [16], [17]. Importantly, inactivation of IAPs does not seem to be detrimental to normal cells. The ensemble of these observations has supported the development of small pharmacological inhibitors of IAPs that have been introduced in phase I clinical trials [5].

LBW242 is a peptidomimetic targeting IAPs recently reported by Zawel and coworkers which competes with high affinity with SMAC/DIABLO for occupancy of the XIAP BIR3 binding pocket [17]. This compound was shown to be able to induce apoptosis of various cell types including multiple myeloma, acute myeloid leukemia, glioblastoma and melanoma [18]–[21].

In the present study we have explored the capacity of LBW242 to induce apoptotic cell death of ovarian cancer cells added alone or in combination with either TRAIL or anticancer drugs. Our results indicate that LBW242 improves the sensitivity of ovarian cancer cell death induced by either TRAIL or anticancer drugs such as Topotecan through an effect related to a potentiation of caspase-8 activation. These observations support future studies to investigate a possible role of LBW242 in ovarian cancer treatment.

**Figure 1:**
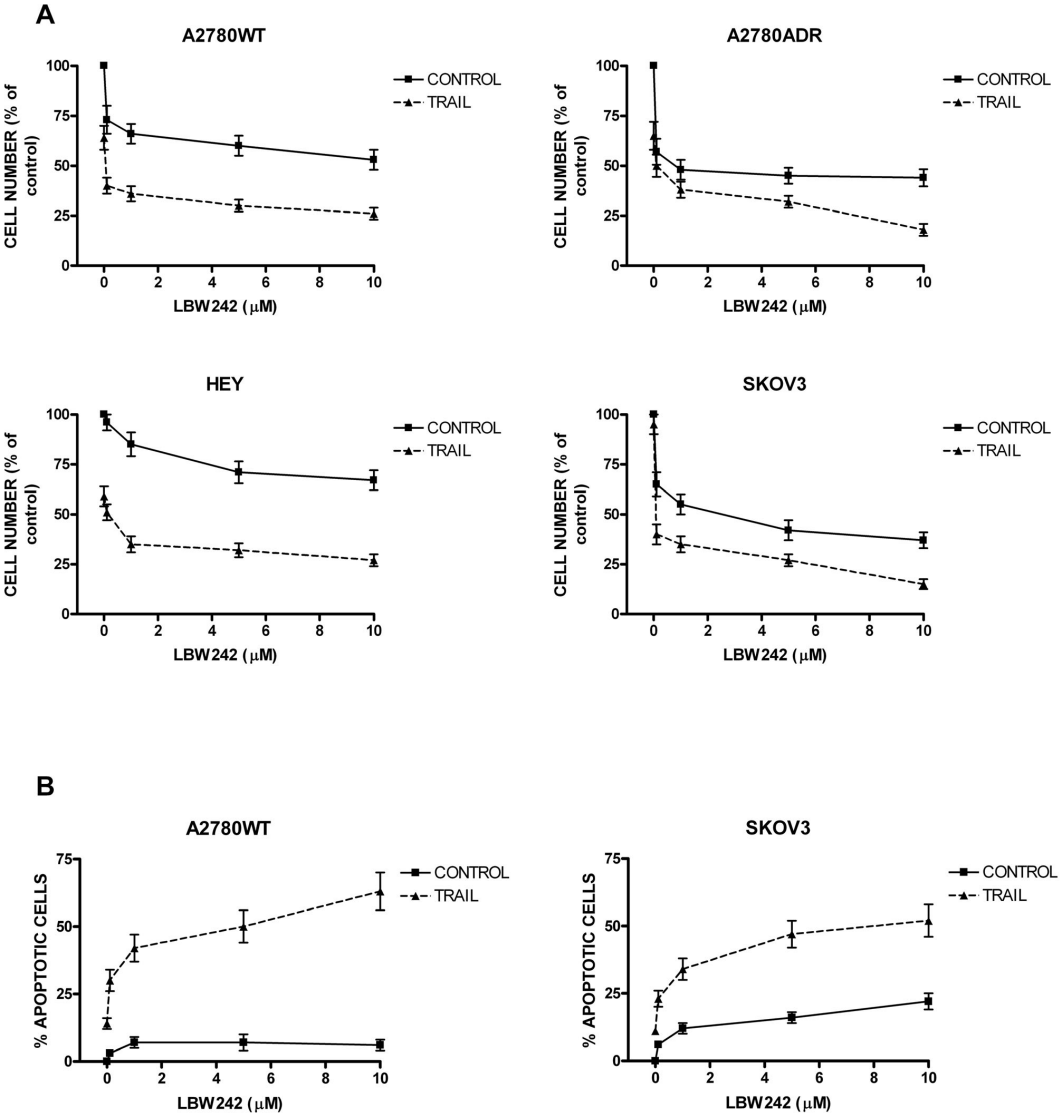
Effect of LBW242 on the cell growth (A) and apoptosis (B) of A2780WT, A2780ADR, HEY and SKOV3 cells. **1A**- A2780WT, A2780ADR, HEY and SKOV3 cells have been grown for 48h either in the absence or in the presence of TRAIL (50 ng/ml) and either in the absence or in the presence of growing concentrations of LBW242 (from 1 to 10 μM) and at the end of the culture the number of living cells was determined. The difference between Control and TRAIL was statistically significant: p = <0.001 for A2780WT and HEY; p = <0.01 for SKOV3; p = <0.05 for A2780ADR. **1B**- A2780WT and SKOV3 cells have been grown as above and after 48 h of culture the proportion of apoptotic cells was determined by Annexin-V binding assay and propide iodide staining. The percentage of apoptotic cells was determined by flow cytometry. The results represent the mean values observed in three separate experiments. The difference between Control and TRAIL was statistically significant: p = <0.01 for both A2780WT and SKOV3 cells.

## Methods

### Ethics statement

This study was specifically approved by the Institutional Review Board of the Istituto Superiore di Sanità and was in accordance with the principles of the Helsinki Declaration II. The written informed content was obtained from each patient.

**Figure 2:**
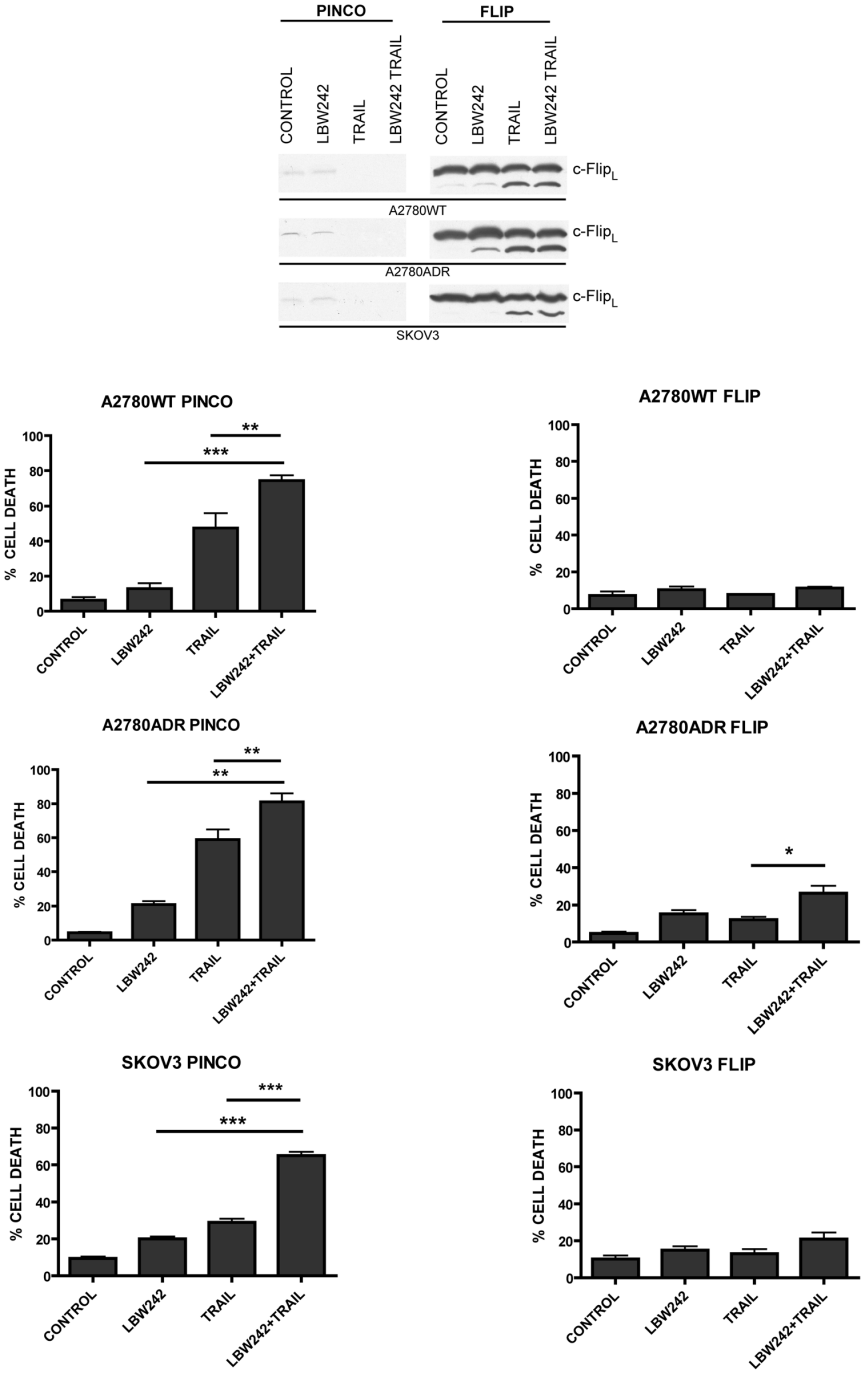
c-FLIP_L_ overexpression protects A2780WT, A2780ADR and SKOV3 cells from the pro-apoptotic effects induced by LBW242. A2780WT, A2780ADR and SKOV3 cells have been stably transfected with either an empty vector (PINCO) or with the vector containing the cDNA of c-FLIP (FLIP) and the resulting cells were grown either in the absence ( Control ) or in the presence of LBW242 (10 μM) or TRAIL (50 ng/ml) or both these agents at the above concentrations. The percentage of apoptotic cells was determined by flow cytometry using the Annexin-V/PI binding assay. The data represent the mean values ± SEM observed in three separate experiments. Statistical analysis: * p = <0.05; ** p = <0.01; *** p = <0.001.

### Cell Culture

Cisplatin-sensitive human ovarian epithelial carcinoma cell line A2780WT was obtained from the American Type Culture Collection (ATCC); adriamycin-resistant cell line A2780ADR, derived from its parental ovarian cancer cell line A2780 by applying stepwise increases in concentrations of adriamycin was obtained from the European Collection of Cell Cultures (ECACC). The A2780ADR cells were treated with 10 µM adriamycin every 10 passages. SKOV3 and HEY cell lines were obtained from ATCC.

**Figure 3:**
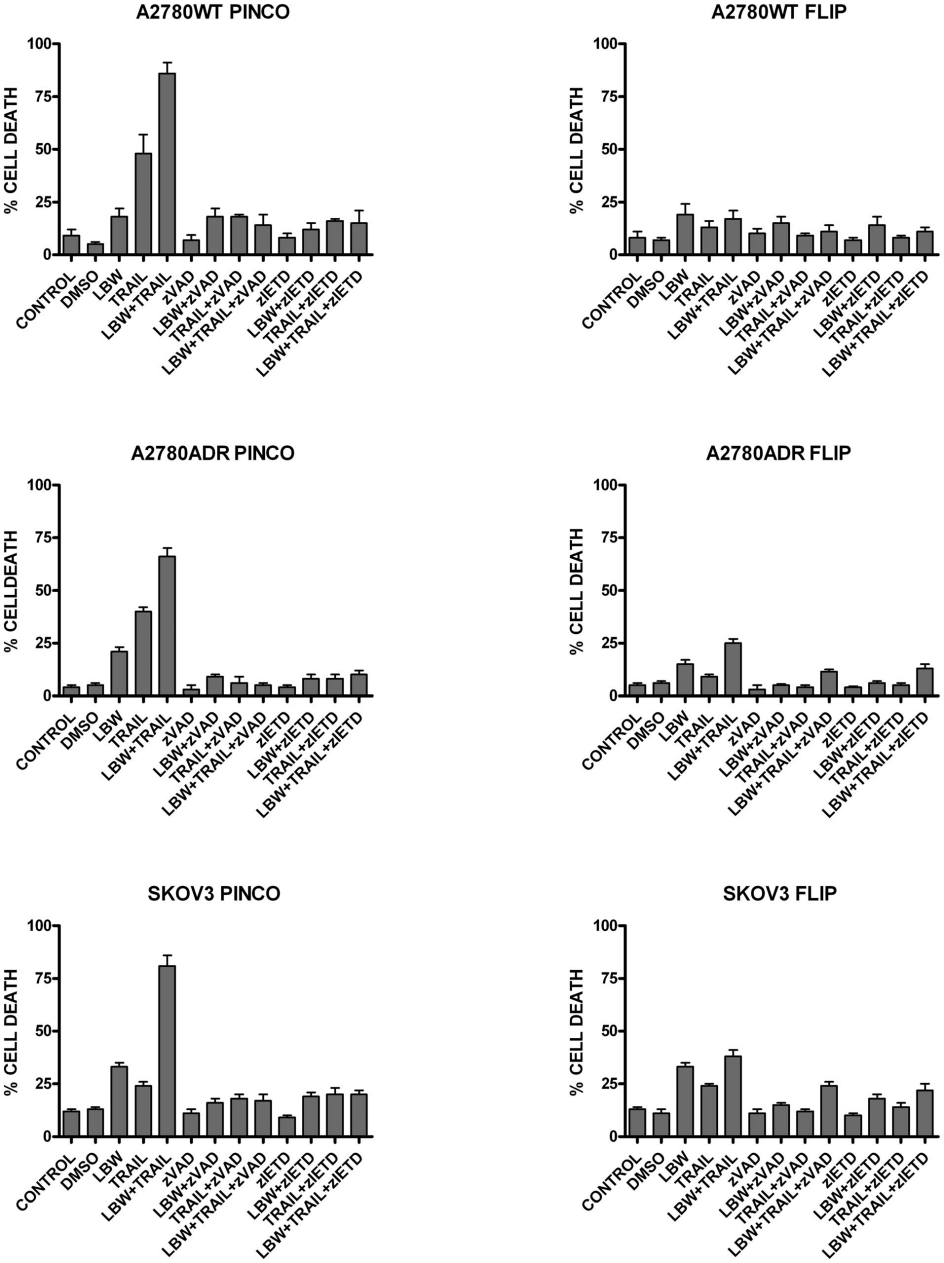
Effect of a pan-caspase inhibitor (zVAD-fmk) and a specific caspase-8 inhibitor (zIETD-fmk) on the induction of apoptosis by LBW242 (LBW) or TRAIL or both agents. A2780WT, A2780FLIP, A2780ADR, A2780ADR FLIP, SKOV3 and SKOV3 FLIP cells have been grown as reported in Fig. 2 either in the absence or in the presence of 40 μM zVAD-fmk or zIETD-fmk and after 48 h of incubation the percentage of apoptotic cells was determined by Annexin-V/PI binding assay. The results represent mean values ± SEM observed in three separate experiments.

The cells were cultured at 37°C in an atmosphere of 5% CO_2_ in Advanced MEM with 3% fetal bovine serum (FBS, Euroclone, Milan, Italy), 50 IU/ml penicillin, 50 µg/ml streptomycin, 50 µg/ml gentamicin and 0,3 µg/ml glutamine. The cells were routinely checked for the presence of mycoplasma.

**Figure 4:**
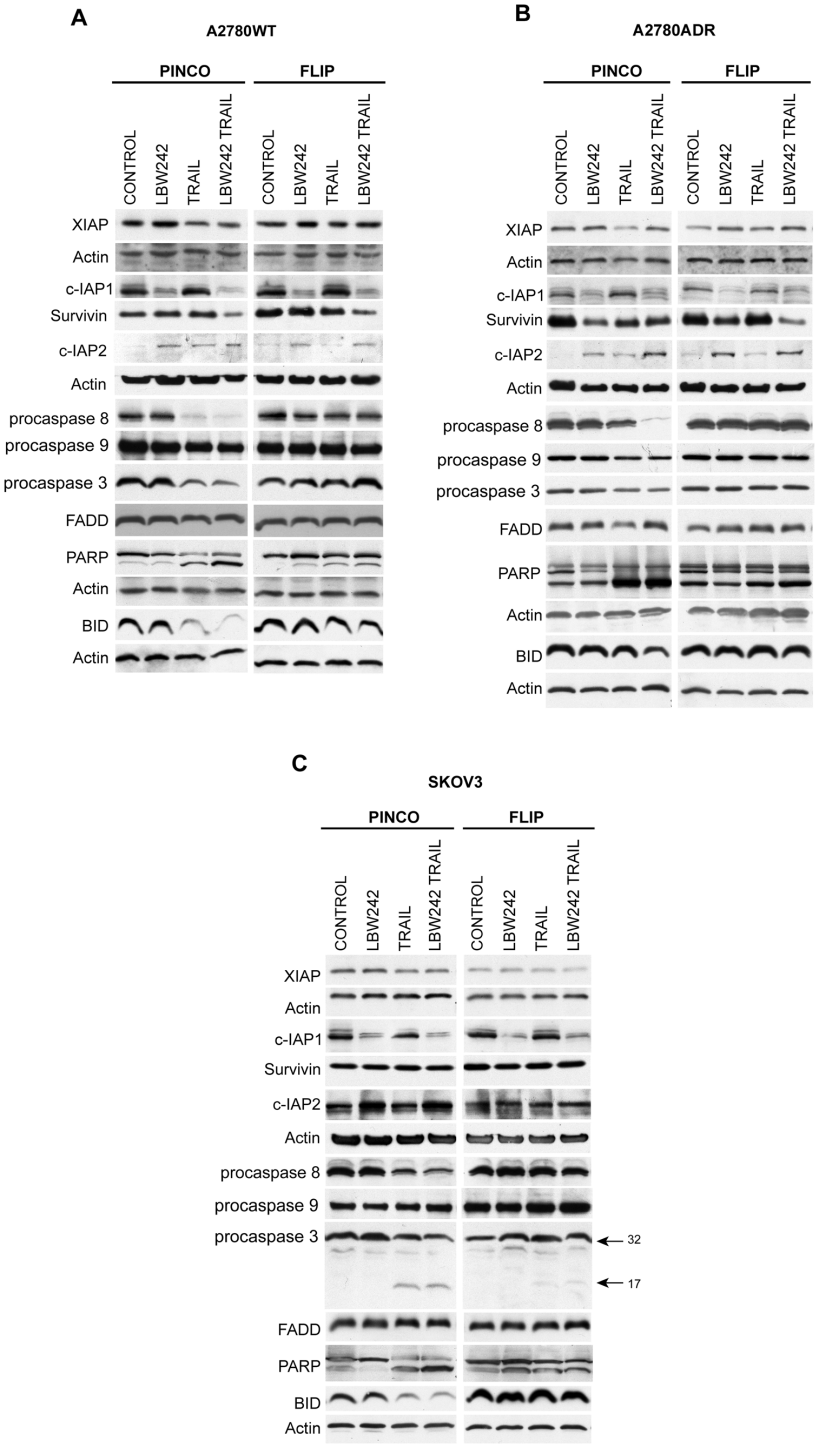
Immunoblotting analysis of XIAP, c-IAP1, c-IAP2, Survivin, Casp-9, Casp-8, c-FLIP_L_, FADD, PARP, Casp-3 and BID in A2780WT PINCO and FLIP (A), A2780ADR PINCO and FLIP (B), SKOV3 PINCO and SKOV3 FLIP (C) cells grown for 24 h either in the absence ( Control ) or in the presence of LBW242 10 μM, or TRAIL 50 ng/ml or both agents at the above concentrations. In the panel C both the full length (FL) and the cleaved form (CF) of caspase-3 are shown. The PARP immunoblotting shows two bands, of which the upper corresponds to a full-length form of PARP and the lower band to a cleaved form of PARP. In the Figure are reported the representative data observed in one of the three experiments carried out.

### Isolation and in vitro culture of primary ovarian cancer cells

Intra-operatory biopsies have been obtained from 9 ovarian cancer patients, affected by serous adenocarcinoma, undergoing debulking surgery for either primary or relapsing disease. Tumor tissue has been mechanically dissociated with a scissor and a tumor cell suspension has been obtained by digestion in tissue culture medium (RPMI 1640) containing collagenase, deoxyribonuclease I and hyaluronidase. The final tumor cell suspension was checked for the proportion of tumor cells by standard cytology and the percentage of epithelial cells by flow cytometry (determined after staining with Ber-EP4 mAb, Dakopatt, Copenaghen, Denmark). Briefly, for the evaluation of Ber-EP4 reactivity cell aliquots were stained 30 min at 4°C with 5 µg/ml FITC-labeled anti-Ber-EP4 mAb, washed and analysed for fluorescence emission using a Becton Dickinson flow cytometer. Tumor cell aliquots (1×10^6^ cells) have been plated into 25 cm^3^ tissue culture flasks in 10 ml of cell culture medium containing 10% fetal calf serum. After 1 day of *in vitro* culture, non-adherent cells (containing tissue debris and dead cells) have been removed and fresh medium was added to the culture and then incubated for additional 24 hours either in the absence or in the presence of TRAIL, or LBW242 or both reagents. At 24 hours of culture cells were confluent. Tumor cultures contained at least 80% of tumor cells.

### Transduction of A2780WT, A2780ADR and SKOV3 cells

A2780WT, A2780ADR and SKOV3 cells expressing either the empty vector PINCO-GFP (PINCO) or the vector PINCO-GFP containing the c-FLIP_L_ (FLIP) human gene have been obtained as previously reported [22]. Transduced cells were routinely analyzed for GFP expression using a flow cytometer and for c-FLIP_L_ expression by Western blotting.

### Apoptosis assessment by Annexin–V staining

After drug treatments, cells were resuspended in 200 µl staining solution (containing Annexin-V fluorescein and Propidium Iodide in a Hepes buffer, Annexin-V-FITC Apoptosis Detection Kit, Pharmingen, San Jose, Ca, USA). Following incubation at room temperature for 15 min., cells were analyzed by flow cytometry. Annexin-V binds to those cells that express phosphatidylserine on the outer layer of the cell membrane, and propidium iodide stains the cellular DNA of those cells with a compromised cell membrane. This allows for the discrimination of live cells (unstained with either fluorochrome) from apoptotic cells (stained only with annexin V) and necrotic cells (stained with both Annexin-V and Propidium Iodide).

### Quantification of apoptosis and cell cycle analysis by propidium iodide/fluorescence activated cell sorting

Cells were harvested with trypsin, washed, incubated first with a spermine tetrahydrochloride detergent buffer containing trypsin to digest cell membranes and cytoskeletons, then with a citrate buffer containing a trypsin inhibitor and ribonuclease A to inhibit trypsin activity and to digest the RNA and, finally, resuspended in 400 µl of propidium iodide (PI) solution (50 µg/ml PI, 0,1% Triton X-100, and 0,1% sodium citrate in PBS) (Cycle Plus DNA Staining Kit, Becton Dickinson, USA). The cells were then analyzed by flow cytometry using a software dedicated for DNA analysis (ModFit LT software, Verity Software House, Topsham, ME, USA). The cells with subdiploid DNA content were quantified to determine the percentage of cells containing apoptotic, fragmented DNA.

### Reagents used to induce apoptosis of tumor cells

Ovarian cancer cells were preincubated with a pan-caspase inhibitor, N-benzyloxy-carbonyl-Val-Ala-Asp(OMe)-fluoromethylketone (zVADfmk) or N-benzyloxy-carbonyl-Ile-Glu(OMe)-Thr-Asp(OMe)-fluoromethylketone (zIETDfmk), (both from Sigma, St Louis, USA) before the addition of the various compounds stimulating apoptosis.

The agonistic monoclonal antibodies to TRAIL-R1 (MAPATUMUMAB) and TRAIL-R2 (LEXATUMUMAB) are fully human antibodies of IgG1 isotype [23], [24] and were generously provided by Human Genome Science (Rockville, MD, USA).

### Treatment with anticancer drugs

In some experiments ovarian cancer cells have been incubated with some anticancer drugs commonly used in ovarian cancer therapy: cis-diamineplatinum(II)chloride (CPLAT); paclitaxel (TAXOL); Topotecan Hydrichloride Hydrate (TOPO); Etoposide (ETOPO); Doxorubicin Hydrochloride (DOXO). All these drugs were purchased from the Sigma Co (St Louis, USA) and were added *in vitro* at two doses: a low dose corresponding to the mean plasma peak level observed during drug infusions to cancer patients (i.e., 1.67 μM for CPLAT; 2.45 μM for TAXOL; 0.21 μM; 8.5 μM for ETOPO; 0.17 μM for DOXO) and a high dose, corresponding to a five fold higher dose than the low dose.

### Western blot Analysis

Whole cell extracts were obtained lysing the cells in a buffer containing 20 mM HEPES, 50 mM NaCl, 10 mM EDTA, 2 mM EGTA, 0.5% NP-40, 1 mM DTT, 0,1 mM PMSF, 2 μg/ml Leupeptin, 2 μg/ml Aprotinin, 25 mM NaF, and 10 mM Na_3_VO_4_. After incubation for 30 min on ice, the protein lysates were cleared of debris by centrifugation at 10,000 g for 10 min. The protein concentration in the soluble supernatant, was determined using the Bio-Rad protein assay (Bio-Rad, Richmond, VA, USA).

**Figure 5:**
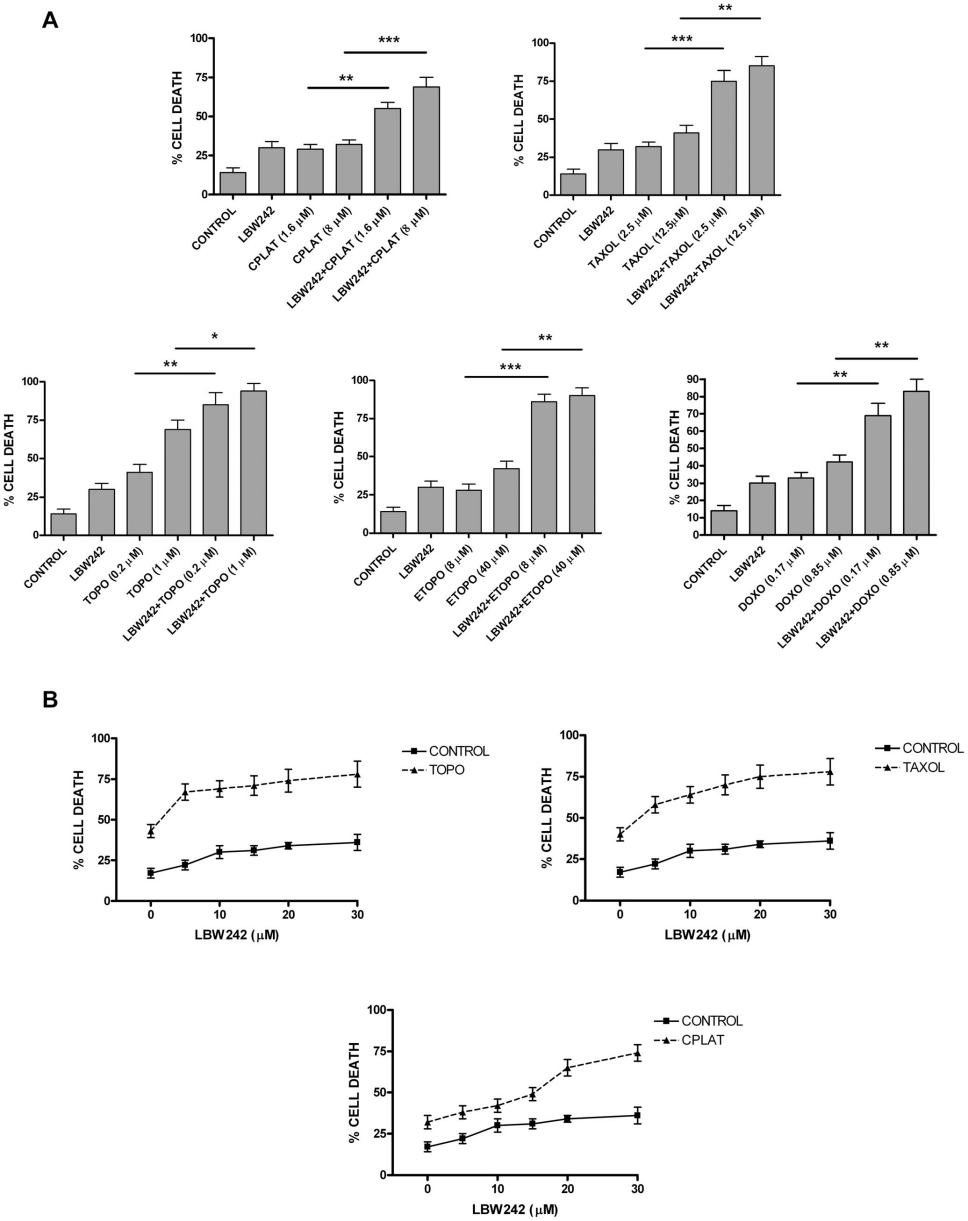
LBW242 potentiates the proapoptotic effects of some anticancer drugs, including Cisplatin, Paclitaxel, Topotecan, Etoposide and Doxorubicin. A- HEY cells have been incubated for 24 h either in the absence (control) or in the presence of LBW242 (30 µM) or of either Cisplatin (CPLAT at 1.6 or 8 µM) or Paclitaxel (TAXOL at 2.5 or 12.5 µM) or Topotecan (TOPO at 0.2 or 1 µM) or Etoposide (ETOPO at 8 or 40 µM) or Doxorubicin (DOXO at 0.17 or 0.85 µM) alone or in combination with LBW242 and analysed for induction of cell death by flow cytometry. The data represent the mean values ± SEM observed in three separate experiments. Statistical analysis: * p = <0.05; ** p = <0.01; *** p = <0.001. B – HEY cells have been incubated for 24 h in the presence of increasing concentrations of LBW242either in the absence (Control) or in the presence of Cisplatin (1.6 µM) or Paclitaxel (2.5 µM) or Topotecan (0.2 µM) and analysed for induction of cell death by flow cytometry. The data represent the mean value ± SEM observed in three separate experiments. The differences between control and Topotecan (p = <0.001), control and Paclitaxel (p = <0.001) and control and Cisplatin (p = <0.05) were all significant.

**Figure 6:**
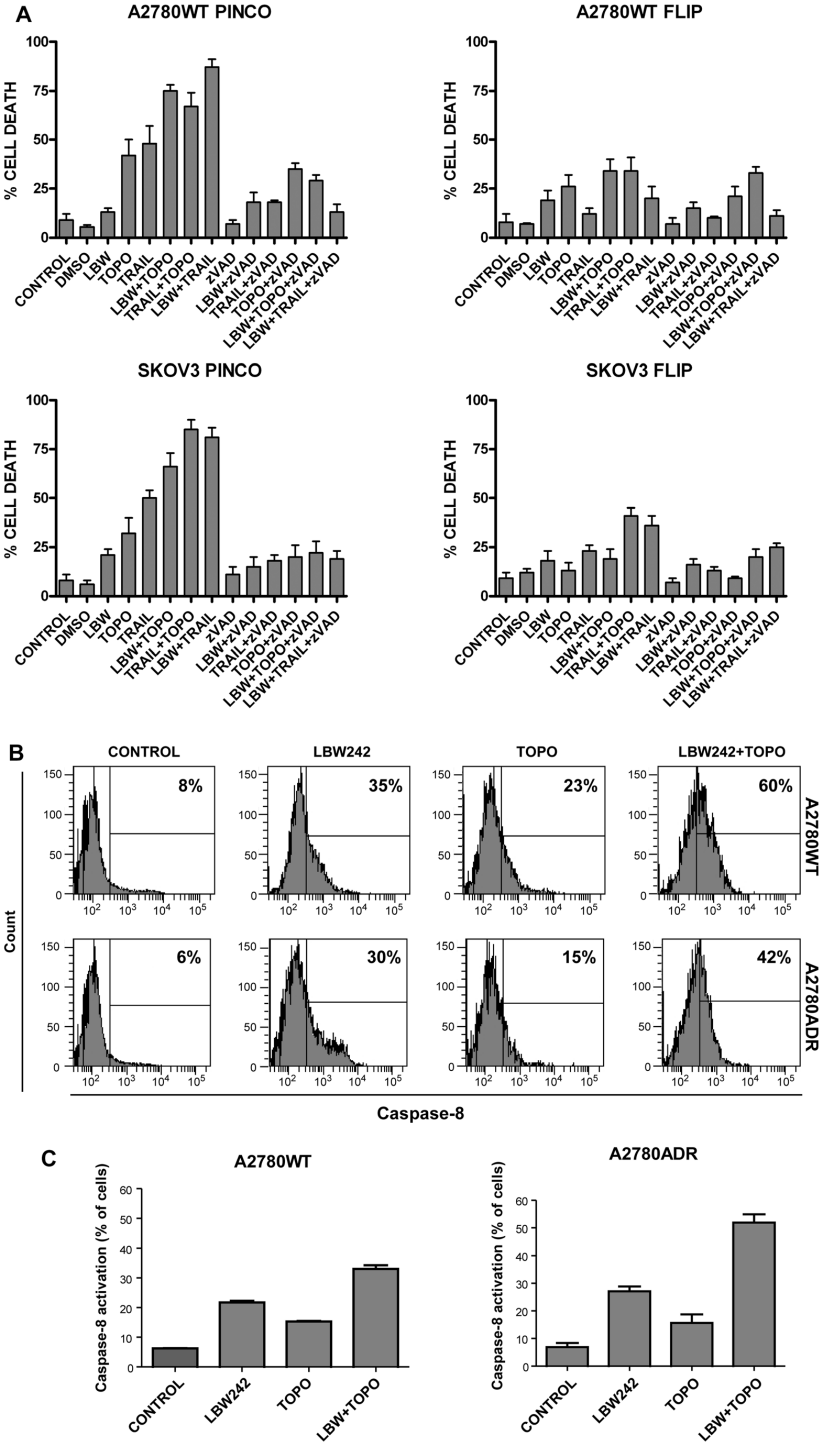
**A**- LBW242 potentiates the proapoptotic effect of Topotecan Hydrochloride, a topoisomerase I inhibitor. A2780WT PINCO and FLIP (*top panels*) and SKOV3 PINCO and FLIP (*bottom panels*) cells have been incubated for 24 h either in the absence (Control) or in the presence of either DMSO 0.01% or zVAD-fmk 40 μM, Topotecan Hydrochloride 1 μM, TRAIL 50 ng/ml or LBW242+zVAD-fmk, LBW242+Topotecan Hydrochloride, TRAIL+zVAD-fmk, TRAIL+Topotecan Hydrochloride, Topotecan Hydrochloride +zVAD-fmk, LBW242+TRAIL, LBW242+TRAIL+zVAD-fmk, LBW242+Topotecan Hydrochloride+zVAD-fmk and analysed for induction of apoptosis by flow cytometry. The data represent the mean values ±SEM observed in three separate experiments. In A7280 WT PINCO and in SKOV3 PINCO cells LBW242+TOPO induced a proportion of dead cells higher than that observed with either LBW242 (p = <0.05) or TOPO (p = <0.05). **B** and **C** – Evaluation of caspases-8 activation in A2780 and A2780ADR cells incubated either in the absence (Control) or in the presence of LBW242 or of Topotecan or of both LBW242 and Topotecan. Caspase-8 activity in intact cells was measured using a caspases-8 specific fluorigenic substrate. Original results from one representative analysis are reported in B, while the mean values±SEM of the percentages of cells displaying caspase-8 activation are reported in C. The percentage of cells exhibiting activated caspases-8 was higher both for WT and ADR cells in LBW242 than in NT cells (p = <0.05) and in LBW242+TOPO than in LBW242 or TOPO-treated cells (p = <0.05).

### Antibodies

Anti-caspase -9, -3 were purchased from Upstate (Upstate Biotechnology Lake Placid NY, USA and R&D Systems Inc. Minneapolis, MN, USA); Anti-XIAP and anti-Bcl-2 were purchased from BD Pharmigen ( BD Pharmigen, San Diego, USA); anti-survivin and anti-PARP were purchased from R&D System (R&D System Inc., Minneapolis, MN); anti-FADD was purchased from BioSource (BioSource, Camarillo, CA, USA); anti-c-FLIP (clone NF6) and anti-c-IAP were purchased from Alexis (Alexis Biochemicals, San Diego, CA, USA); anti-actin from Oncogene (Oncogene research Products, Cambridge, MA), was used as loading control.

### Statistical Analysis

Statistical analysis was performed using the Graph Pad Program. All parameters were reported as means ± SEM. To compare between group differences, robust ANOVA was performed. The P-values reported were two-sided. A P-value of less than 0.05 indicated statistical significance. Isobologram analysis was performed using the CalcuSyn software program (Biosoft, MO, and Cambridge, UK). A combination index (CI) less than 1.0 indicates synergism, an a CI of 1.0 indicates additive activity [25].

## Results

### LBW242 improves TRAIL-mediated cell death of ovarian cancer cell lines

In our previous studies we showed the pro-apoptotic effect of SMAC/DIABLO mimetic compound 3 in ovarian cancer cell lines A2780WT and their resistant counterpart A2780DDP and A2780ADR [26]. In the present study, we investigated the effect of another SMAC/DIABLO mimetic, LBW242, on ovarian cancer models. First, we explored the cell proliferation in a dose-response test of LBW242 alone and in combination with TRAIL (FIG. 1A). The cell line A2780WT (*upper left panel*) was only slightly inhibited in its growth by LBW242 added alone; however, the combined treatment of LBW242 with TRAIL resulted in a marked synergistic inhibition of cell growth. The same kind of sensitivity was observed for HEY cell line (*lower left panel*). Isobologram analysis confirmed synergistic anti-tumor activity of LBW242 plus TRAIL (for A2780WT CI = 0.77 and HEY CI = 0.80). The synergistic interaction of LBW242 and TRAIL in A2780WT and HEY cell lines can be intuitively visualized by plotting cell growth data using measurements at LBW242 0 μM with and without TRAIL as 100% and all the subsequent measurements expressed relative to its own control (see Fig. S1). This figure reveals an additional reduction in cell number caused by the addition of LBW242 on the top of that induced by TRAIL alone (Fig. S1).

**Figure 7:**
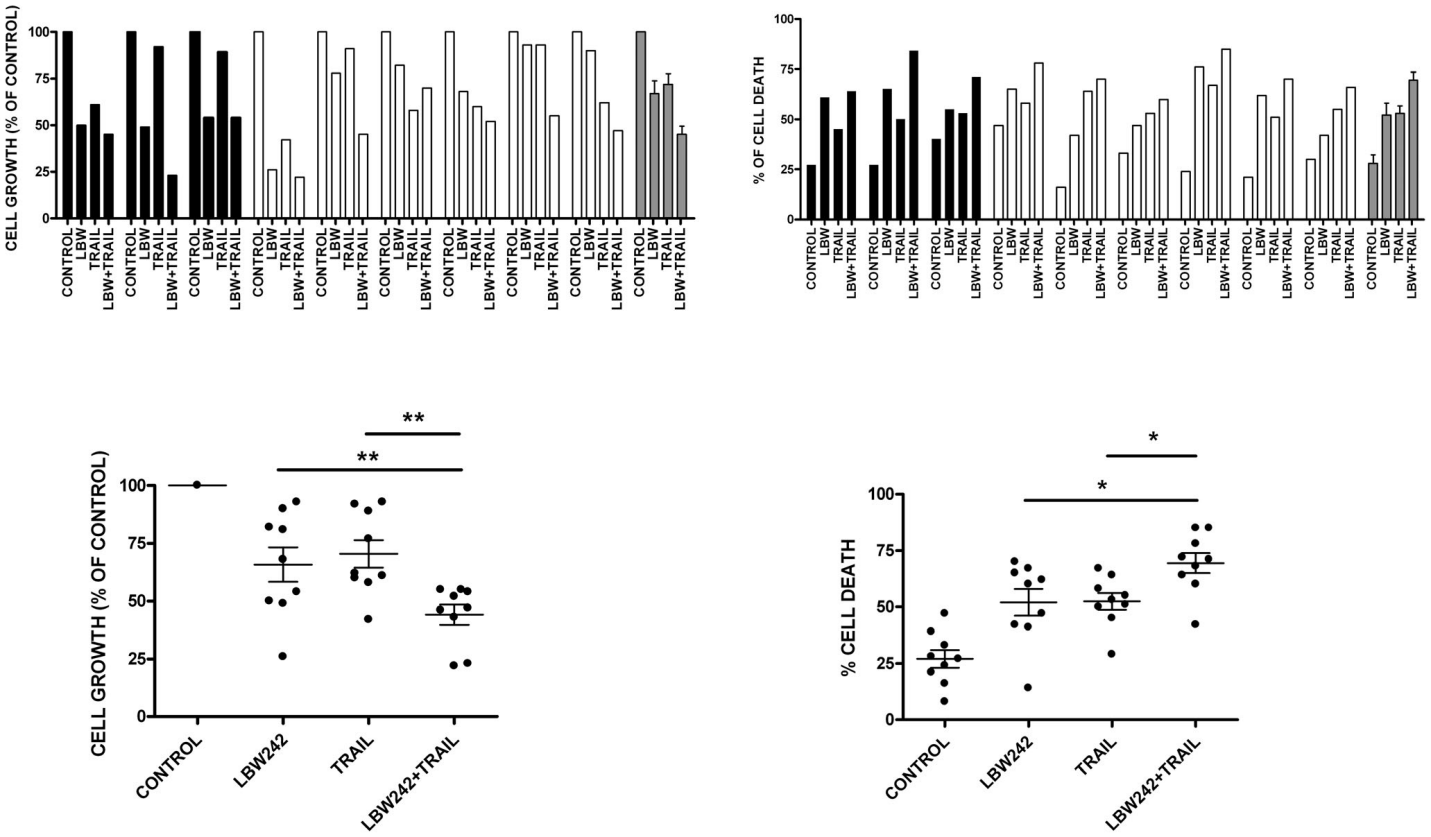
Effect of LBW242, TRAIL or a combination of both agents on cell growth (*left upper panel*) and induction of apoptosis (*right upper panel*) of ovarian cancer cells derived from 3 ovarian cancer at presentation (black bars) and 6 relapsing ovarian cancers (white bars). The gray bars represent mean values ± SEM. Ovarian cancer cells isolated from tumor biopsies have been grown for 24 hours either in the absence or in the presence of LBW242 (10 μM) or of TRAIL (50 ng/ml) or both agents at the above concentrations. *Lower panel on the right*: The proportion of dead cells was higher in LBW242 or TRAIL or LBW242+TRAIL-treated cells than in control not-treated cells (p = <0.01); furthermore, the percentage of dead cells was lower in LBW242+TRAIL than in TRAIL or LBW242-treated cells (for both p = <0.05). *Lower panel on the left*: The cell number was significantly lower in LBW242+TRAIL than in TRAIL or LBW242-treated cells (for both p = <0.05).

**Table 1:** Clinical characteristics of the nine ovarian cancer patients whose tumor samples were Investigated for sensitivity to LBW242.

PATIENT	AGE	Clinical Stage	FIGO Stage	Pathology	Tumor Site	Therapy
1	62	Primary	III c	Scarcely differentiated serous carcinoma	Peritoneum	3c Carboplatin-Taxol
2	81	Primary	I a	Scarcely differentiated endometrioid carcinoma	Ovary	6c Carboplatin-Taxol
3	39	Primary	III c	Serous carcinoma	Ovary	3c Carboplatin-Taxol
4	81	Relapse	III c	Serous carcinoma	Peritoneum	6c Carboplatin-Taxol
5	71	Relapse	III c	Serous carcinoma	Omentum + Peritoneum	6c Carboplatin- Taxol
6	59	Relapse	IV	ADC-Serous carcinoma	Omentum	5c Cisplatin- Farmoribicin-Taxol
7	60	Primary	III c	Serous carcinoma	Ovary	3c Carboplatin-Taxol
8	41	Relapse	III c	Serous carcinoma	Peritoneum	8c Paclitaxel- carboplatin
9	55	Relapse	III c	Papillary-cystic carcinoma	Omentum	8c Carbo-taxol

The A2780ADR and SKOV3 (*right panels*) cell lines were the most sensible to the inhibitory effect of LBW242 on cell proliferation, which was moderately increased by TRAIL addition. Isobologram analysis showed an additive effect of LBW242 plus TRAIL in inhibiting the growth of these cell lines (for A2780ADR CI = 0.97; for SKOV3 CI = 1.0).

The same treatments were performed on A2780WT and SKOV3 cell lines to evaluate the effect of LBW242 on the percentage of apoptotic cells (Fig. 1B). The A2780WT cells were scarcely sensitive to the single treatments with either LBW242 or TRAIL alone, but very sensitive to the combined treatment (CI = 0.70). SKOV3 cells were sensitive to the pro-apoptotic effect of LBW242, but scarcely sensitive to TRAIL; the combined addition of the two drugs further increased the rate of apoptosis (CI = 0.79) (Fig. 1B).

These data indicate that: ovarian cancer cell lines are sensitive to LBW242 effects, particularly in combined treatment with TRAIL; LBW242 exerts a synergistic or additive anti-tumor activity with TRAIL in ovarian cancer cell lines.

Experiments carried out using agonistic anti-TRAIL-R1 (MAPATUMUMAB) or anti-TRAIL-R2 (LEXATUMUMAB) mAbs provided evidence that the latter one added together with LBW242 induced a high rate of apoptosis of all the four ovarian cancer cell lines here studied (data not shown).

### c-FLIP_L_ overexpression inhibits the pro-apoptotic effect of LBW242

In previous studies it was demonstrated that under TNFα stimulation, caspase-8 is a critical apoptotic protease in IAP antagonist-induced cell death [27]–[30]. To explore a possible role of caspase-8 activation in LBW242-mediated cell death, we used cell lines stably transfected with c-FLIP_L_ (A2780WT FLIP, A2780ADR FLIP and SKOV3 FLIP), a natural caspase-8 inhibitor. A2780WT, A2780ADR and SKOV3 cells express low levels of c-FLIP, c-FLIP_L_ being the only isoform detectable in these cells (Fig. 2 and Fig. S2). In contrast, as it is expected, A2780WT FLIP, A2780ADR FLIP and SKOV3 FLIP express high levels of c-FLIP_L_ (Fig. 2 and Fig. S2). C-FLIP_S_ was undetectable in all these cell lines (Fig. S2).

Notably, in A2780WT, ADR and SKOV3 cells (Fig. 2, *left panel* *s*) transfected with empty vector (PINCO), the single treatment with LBW242 or TRAIL induces a moderate apoptotic effect, while the combined treatment of LBW242 with TRAIL induces a remarkable increase in cell death. In those cells overexpressing c-FLIP_L_ (Fig. 2, *right panel* *s*) the effect of LBW242 treatment alone or in combination with TRAIL is highly inhibited, thus supporting the hypothesis that SMAC/DIABLO mimetic may act through induction of a caspase-8 activation pathway.

To this end cells were also treated with a pan-caspase inhibitor zVAD, or with a specific caspase-8 inhibitor, zIETD; in Fig. 3 the percentage of cell death was reported. As expected zVAD protects cells from the apoptotic effect of both single and combination treatments, thus indicating caspase activation involvement in LBW 242 + TRAIL-mediated cell death. Moreover, it is possible to note that the effect of zIETD is comparable to that of zVAD, thus indicating a key role of caspase-8 activation under the combined action of LBW242 and TRAIL treatments (Fig. 3, *left panel* *s*).

### LBW242 added together with TRAIL induced caspase-8 activation

Biochemical analyses were performed by investigating the expression of different proteins involved in the apoptotic pathway in A2780WT PINCO, A2780ADR PINCO, SKOV3 PINCO and A2780WT FLIP, A2780ADR FLIP and SKOV FLIP cell lines treated as above mentioned. In Fig. 4 A, B and C after 24 h treatments, it is possible to note that XIAP levels are not affected by LBW242. This finding is in line with previous studies showing that LBW242 inhibits XIAP activity, without affecting its expression [17]–[21]. As expected on the basis of previous reports [17]–[21], LBW242 dramatically downmodulates c-IAP1 levels, a phenomenon clearly seen in all cell lines, including those overexpressing c-FLIP_L_. Treatment of ovarian cancer cell lines with LBW242 induced an effect on c-IAP2 expression very different from that observed for c-IAP1. In fact, 24 h exposure of the cells to LBW242 induced a clear upmodulation of c-IAP2 levels (Fig. 4), in line with a previous report [31]. As it is expected, c-FLIP_L_ overexpression did not modify the effect of LBW242 on c-IAP2 levels (Fig. 4). On the other hand, TRAIL induced a moderate increase of c-IAP2, a finding in line with previous studies [32]. Notably, c-FLIP overexpression prevented the stimulatory effect of TRAIL on c-IAP2 levels (Fig. 4). The combined treatment with LBW242 and TRAIL resulted in a marked upmodulation of c-IAP2 levels (Fig. 4). Furthermore, LBW242 failed to induce either caspase-8, caspase-9 or caspase-3 activation, as it can be inferred from the absence of any significant reduction of procaspase-8, -9 and -3 levels and of the appearance of active caspase cleaved fragments (see Fig. 4 A to C and Fig. S3). In line with these findings, LBW242 failed to induce cleavage of PARP, a sensitive target of activated caspase-3 (Fig 4A to C).

When LBW242 was added together with TRAIL markedly potentiated the effect of this death ligand on caspase-8 and caspase-3 activation and on PARP cleavage in A2780 WT, A2780 ADR and SKOV 3 cell lines transfected with the empty vectors, but not in those overexpressing c-FLIP_L_ (Fig. 4 A to C). It is important to note that caspase-8 activation, triggered by TRAIL and mostly by LBW242+TRAIL, leads to BID degradation, thus achieving the activation of the intrinsic apoptotic pathway (Fig. 4 A to C). Bid degradation was almost completely prevented by c-FLIP overexpression (Fig. 4 A to C).

### LBW242 improved the pro-apoptotic effect of anticancer drugs on ovarian cancer cells

The standard medical treatment of ovarian cancer involves in first instance the use of platin and taxol anticancer drugs. Therefore, it seemed of interest to evaluate a possible cooperative effect of some anticancer drugs, such as cisplatin, taxol, topotecan, etoposide and doxorubicin in combination with LBW242. Thus, in a first set of experiments we treated HEY cells with Cisplatin or Paclitaxel or Topotecan or Etoposide or Doxorubicin, added at two doses, a lower clinical dose corresponding to the peak dose observed during infusion of these drugs to cancer patients and a 5 times higher supraclinical dose, alone or in combination with a plateau dose of LBW242 (30 μM). The results of this experiment clearly showed that LBW242 was able to potentiate the cytotoxic effects of all these drugs (Fig. 5A). Isobologram analysis showed synergistic anti-tumor activity of LBW242 plus Cisplatin (CI = 0.82) or plus Paclitaxel (CI = 0.70) or plus Topotecan (0.72) or plus Etoposide (CI = 0.73) or plus Doxorubicin (p = 0.78). In a second experiment we tested the capacity of various doses of LBW242 to enhance the cytotoxicity induced by a clinical dose of Cisplatin, Taxol or Topotecan. This experiment showed that LBW242 in a dose-dependent manner increased the cytotoxic response to these drugs, achieving the maximal effects at 20–30 μM dosages (Fig. 5B). Isobologram analysis showed synergistic anti-tumor activity of LBW242 plus Topotecan (CI = 0.70) or Taxol (CI = 0.71) or Cisplatin (CI = 0.82).

Based on these findings, in a second set of experiments we focused our attention on the capacity of LBW242 to enhance the effects of Topotecan, a drug used in ovarian cancer treatment and known as a potential caspase-8 activator [33].

In Fig. 6A it is possible to note that Topotecan exerts a marked pro-apoptotic effect when added in combination with LBW242 and TRAIL in A2780WT PINCO cells; the cell death of tumor cells caused by these agents when used in combination is greatly inhibited by the pan-caspase inhibitor zVAD-fmk and therefore is mainly mediated through caspase activation.

In A2780WT FLIP cells the apoptotic induction triggered by the association between LBW242, TRAIL and Topotecan is almost completely inhibited, thus suggesting a major role of caspase-8 in the induction of tumor cell death (Fig. 6A).

These observations have been confirmed in SKOV3 PINCO and SKOV3 FLIP cells (Fig. 6A). A role for caspases-8 activation in Topotecan-induced death of ovarian cancer cells was further supported by experiments of quantification of caspases-8 activation. These experiments showed that both in A2780WT and A2780ADR cells either LBW242 or Topotecan alone induced caspase-8 activation in a few percentage of cells; however, the two compounds added together resulted in the caspase-8 activation in a high percentage of cells (Fig. 6B and C).

### LBW242 markedly potentiates TRAIL-mediated apoptosis of primary ovarian cancer cells

Given the results obtained in ovarian cancer cell lines we have explored whether LBW242 could induce, when added alone or in combination with TRAIL, apoptosis of primary ovarian cancer cells. To perform this analysis primary ovarian cancer cells derived from human tumor biopsies obtained during surgery debulking of tumor mass of 3 primary and 6 relapsing ovarian cancers have been isolated and tested for their sensitivity to LBW242. The clinico-pathological features of these patients are reported in Table 1. The analysis of the percentage of apoptotic cells and of the number of living cells was carried out. The nine cases analyzed displayed a heterogeneous sensitivity to TRAIL, with the majority of cases (about 60%) displaying a low sensitivity to the apoptotic effect of this death ligand (Fig. 7). A similar situation was observed for LBW242, with about 50% of the samples displaying a clear sensitivity to the SMAC/DIABLO mimetic (Fig. 7). Interestingly, all these samples displayed also a moderate sensitivity to TRAIL. The contemporaneous treatment with LBW242 and TRAIL increased the rate of apoptosis of ovarian cancer cells compared to the values observed when the cells were grown with TRAIL alone (p<0.05) or LBW242 alone (p<0.05). Importantly, relapsing ovarian cancer cells, that are usually chemoresistant displayed a clear sensitivity to apoptotic triggering by LBW242+TRAIL addition as well as primary ovarian cancer samples.

When the number of living cells was analyzed we observed that either LBW242 or TRAIL added alone induced a moderate decline of the number of living cells, while the decline was clearly more pronounced when these two agents were added together (Fig. 7).

## Discussion

Previous studies based on the enforced expression of SMAC/DIABLO into ovarian cancer cell lines have provided initial evidences indicating that through this way apoptosis can be induced in ovarian carcinoma without damaging normal ovarian tissue [], [35]. Alternatively, apoptosis of ovarian cancer cells can be induced using small peptides, such as compound 3, that act as SMAC/DIABLO mimetics [24]. Recently, more potent IAP inhibitors were synthesized and some of them, like LBW242, entered phase I clinical studies [17]–[21].

It is of fundamental importance in the studies on these compounds, as well as on other new anticancer drugs, to identify candidate cancer types suitable for subsequent clinical studies. Given our previous studies with the compound 3 SMAC/DIABLO mimetic on ovarian cancer, it seemed particularly interesting to extend these studies to LBW242. The main objectives of the present study consisted in determining: (a) the sensitivity of ovarian cancer cell lines and particularly of those chemoresistant, as well as of primary ovarian cancer cells, to LBW242-mediated apoptosis; (b) whether the treatment with LBW242 sensitizes ovarian cancer cells to TRAIL-mediated apoptosis; (c) whether LBW242 potentiates the cytotoxic effects of some anticancer drugs used in the treatment of ovarian cancer.

Concerning the first point, we observed that treatment of ovarian cancer cells with LBW242 resulted in a moderate inhibitory effect on cell proliferation, in part due to induction of apoptosis of these cells. These observations were in line with previous studies carried out on other cancer cell types [17]–[21]. It is important to note that LBW242 was equally active in inhibiting the growth of both chemosensitive and chemoresistant ovarian cancer cell lines, as well as of fresh ovarian cancer cells derived from primary and relapsing tumors.

Concerning the second point, we observed that LBW242, like SMAC/DIABLO mimetic compound 3 [24], sensitized ovarian cancer cells to the pro-apoptotic effects of TRAIL. These observations are potentially interesting because they provided evidence that the combined addition of LBW242 and TRAIL resulted in the massive cell death of all ovarian cancer cell types here analyzed, including those derived from relapsing ovarian cancer patients. The potentiation of TRAIL-mediated apoptosis by LBW242 seems to be related to the capacity of IAP inhibitors to favor caspase-8 activation, as previously indicated in various reports [27]–[30]. In particular, using other SMAC/DIABLO mimetics, it was shown that c-IAP1 degradation induced by the mimetics leads to formation of caspase-8 activation complex involving procaspase-8, FADD and RIP1, RIP1 being required for pro-apoptotic activity [36]–[37]. In our experiments, this conclusion is supported by various lines of evidence: (i) overexpression of c-FLIP_L_, a natural caspase-8 inhibitor, in ovarian cancer cell lines resulted in a protection against the pro-apoptotic effects elicited by LBW242 alone or in combination with TRAIL; (ii) addition of a synthetic cell-permeable caspase-8 inhibitor, zIETD-fmk, to ovarian cancer cells resulted in a marked inhibition of LBW242+TRAIL-induced apoptosis. These findings substantiate the essential role of caspase-8 in tumor cell apoptosis elicited by IAP inhibitors. It is important to note that a previous study showed that down-regulation of c-FLIP in tumor cells including ovarian cancer, enhances apoptosis induced by SMAC/DIABLO mimetic compounds [38]. In this context, it is of interest to note that recent studies suggest an important role for a caspase-8 gene polymorphism in the susceptibility to develop ovarian cancer and breast cancer [39], [40].

Previous studies have shown that some cancer cell lines evade Smac mimetic-induced apoptosis by upregulating c-IAP2, which although initially degraded, rebounds and it is refractory to subsequent degradation [31], [41]. We confirmed this phenomenon of c-IAP2 upmodulation by LBW242. C-IAP2 upmodulation induced by LBW242 in ovarian cancer cells could induce some resistance to apoptosis; however, this inhibition was overcome by the addition of TRAIL to the SMAC/DIABLO inhibitor.

Concerning the third point, we have explored a possible potentiating effect of LBW242 on tumor cell death induced by some anticancer drugs. Particularly, we described that LBW242 synergizes with Cisplatin, Taxol, Topotecan, Etoposide and Doxorubicin in inducing ovarian cancer cell death. It is important to point out that this stimulatory effect of LBW242 on the cytotoxic effects mediated by anticancer drugs is exerted both on drugs used in first line (cisplatin and taxol) and in second line (topotecan, etoposide and doxorubicin) treatments. These observations are in line with previous studies providing evidence about a potentiation of antileukemic on anti-multiple myeloma therapies by LBW242 [18], [19]. In this context, particularly interesting was a recent report showing that LBW242 enhanced esophageal squamous cell carcinoma cisplatin-induced apoptosis and caspase activation and restored cisplatin sensitivity in SMAC-deficient tumor cells [42].

We observed that LBW242 clearly potentiated the *in vitro* antitumor effects of Topotecan, a topoisomerase I inhibitor, known as a potential caspase-8 activator [31]. The cell death induced by LBW242+Topotecan Hydrochloride was at large extent inhibited by either c-FLIP_L_ overexpression or zIETD-fmk, thus suggesting that the two drugs cooperate in inducing caspase-8 activation. Interestingly, we observed also that LBW242 potentiated the cytotoxic effect of Etoposide. This finding may be related to the different capacity of these two compounds to mediate the degradation of IAP machinery, Etoposide being capable of inducing c-IAP1, c-IAP2, XIAP and, most importantly c-FLIP_L_ degradation, as shown by a recent study [43].

In conclusion, the results of the present study indicate that the IAP inhibitor LBW242 is active in mediating inhibition of ovarian cancer cells, particularly when acting in combination with TRAIL or with some antineoplastic drugs. These observations may represent the preclinical rationale for future experimental studies of small molecule inhibitors in combination with TRAIL or with some antineoplastic drugs.

## Supporting Information

Figure S1 **Effect of LBW242 on the cell growth of A2780WT, A2780ADR, HEY and SKOV3 cells.** The data are identical to those reported in Fig. 1A, but are plotted using measurements at LBW242 0 μM with and without TRAIL as 100% and all the subsequent measurements expressed relative to its own control.
(TIF)

Figure S2 **Immunoblotting analysis of c-FLIP in A2780WT PINCO and FLIP (A), A2780ADR PINCO and FLIP (B), SKOV3 PINCO and SKOV3 FLIP (C) cells grown for 24 h either in the absence ( Control ) or in the presence of LBW242 10 μM, or TRAIL 50 ng/ml or both agents at the above concentrations.**
(TIF)

Figure S3 **Immunoblotting analysis of Casp-3, Casp-8 and Casp-9 in A2780WT PINCO and FLIP (A), A2780ADR PINCO and FLIP (B), SKOV3 PINCO and SKOV3 FLIP (C) cells grown for 24 h either in the absence ( Control ) or in the presence of LBW242 10 μM, or TRAIL 50 ng/ml or both agents at the above concentrations.** The cleavage bands of 43/41 kDa for caspase-8, of 37/35 kDa for caspase-9 and of 17 kDa for caspase-3 are shown.
(TIF)
