# Supplementary figures and images for: A Small Molecule SMAC Mimic LBW242 Potentiates TRAIL- and Anticancer Drug-Mediated Cell Death of Ovarian Cancer Cells
Source: PLoS One. 2012 Apr 25;7(4):e35073. doi: 10.1371/journal.pone.0035073 (PMC3338831; doi:10.1371/journal.pone.0035073)

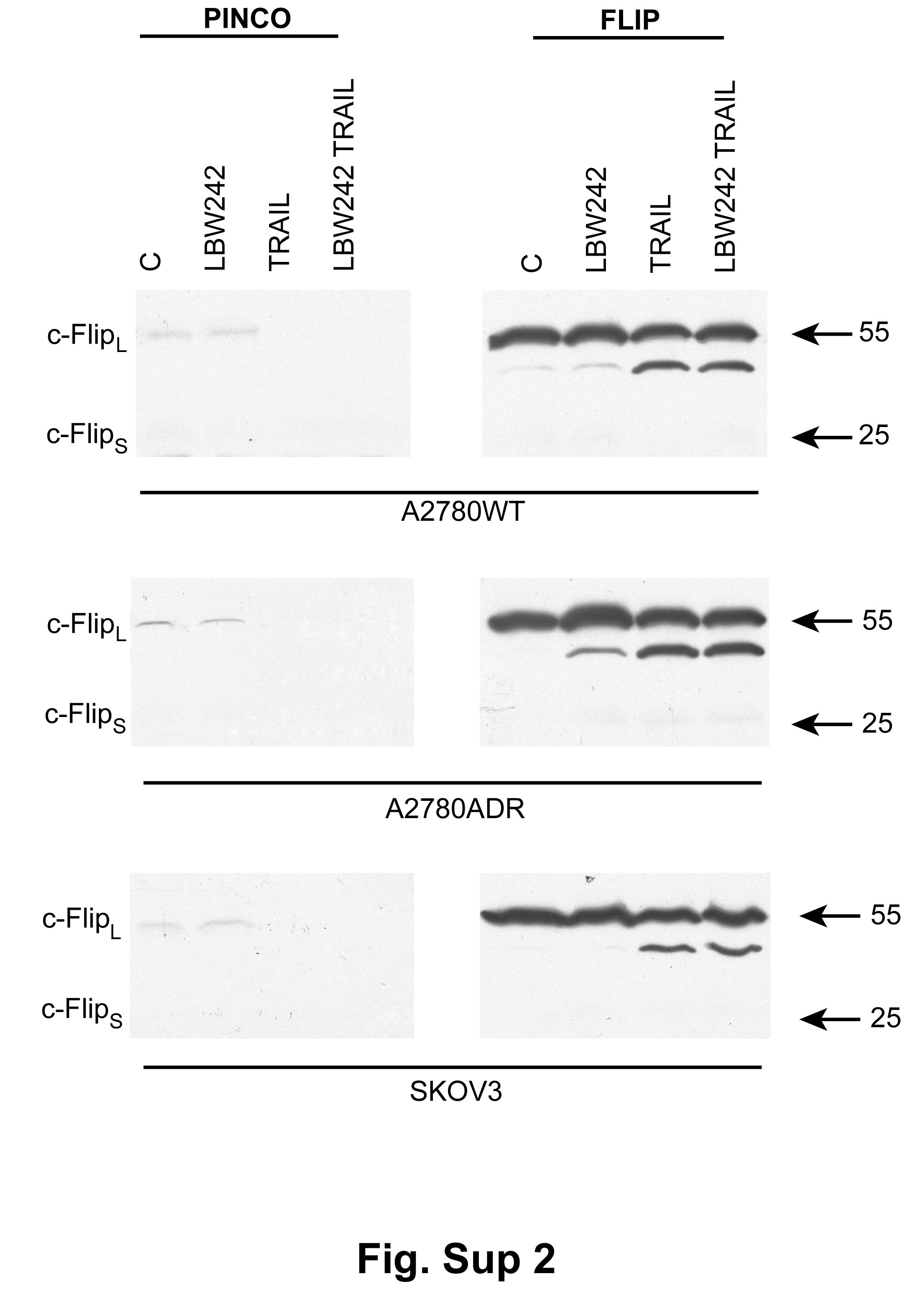

Supplement: Figure S2 — Immunoblotting analysis of c-FLIP in A2780WT PINCO and FLIP (A), A2780ADR PINCO and FLIP (B), SKOV3 PINCO and SKOV3 FLIP (C) cells grown for 24 h either in the absence ( Control ) or in the presence of LBW242 10 μM, or TRAIL 50 ng/ml or both agents at the above concentrations. (TIF) [file pone.0035073.s002.tif]

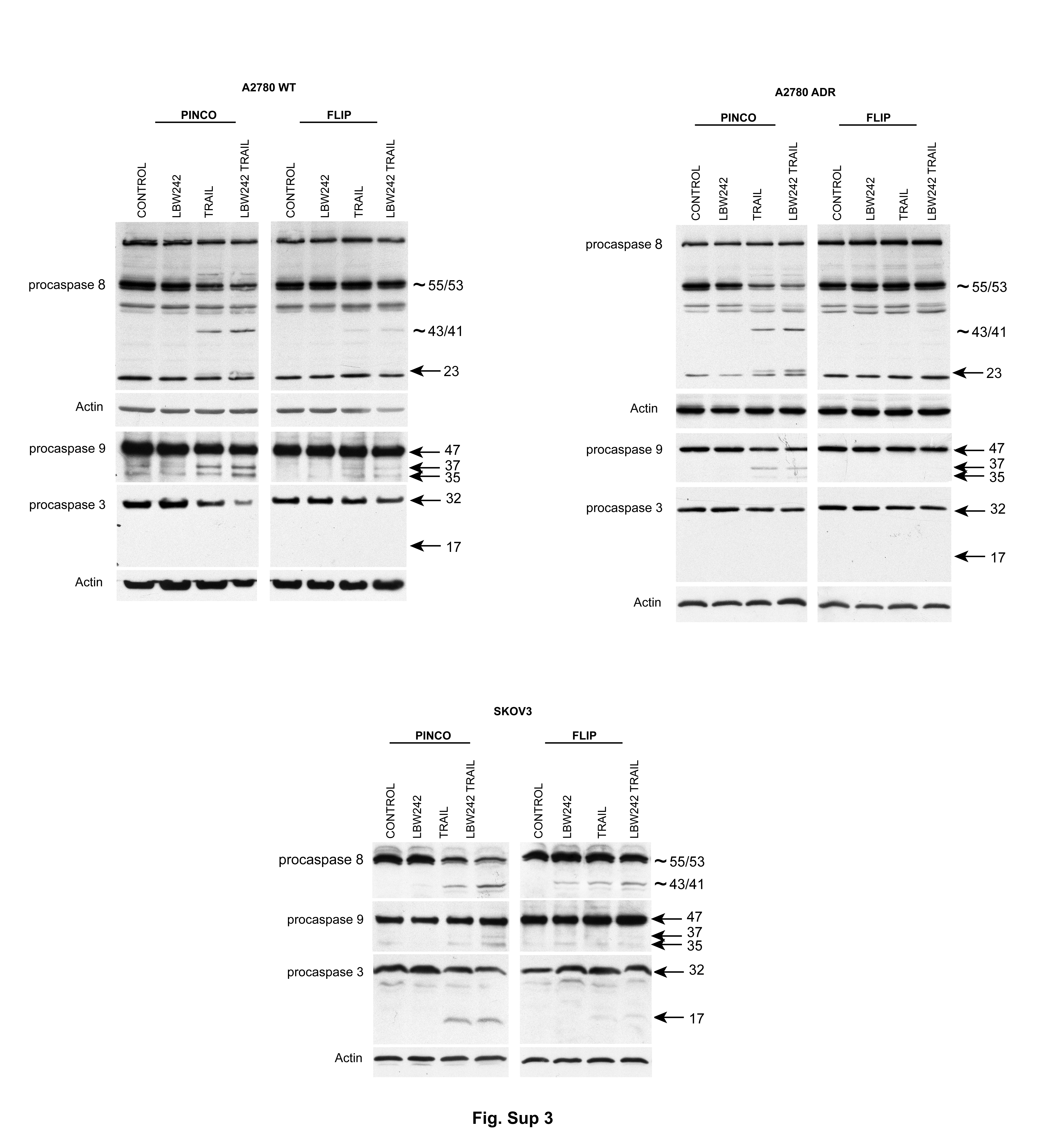

Supplement: Figure S3 — Immunoblotting analysis of Casp-3, Casp-8 and Casp-9 in A2780WT PINCO and FLIP (A), A2780ADR PINCO and FLIP (B), SKOV3 PINCO and SKOV3 FLIP (C) cells grown for 24 h either in the absence ( Control ) or in the presence of LBW242 10 μM, or TRAIL 50 ng/ml or both agents at the above concentrations. The cleavage bands of 43/41 kDa for caspase-8, of 37/35 kDa for caspase-9 and of 17 kDa for caspase-3 are shown. (TIF) [file pone.0035073.s003.tif]
